# Supplementary material for: Transcriptome Analysis Reveals Vimentin-Induced Disruption of Cell–Cell Associations Augments Breast Cancer Cell Migration
Source: Cells. 2022 Dec 13;11(24):4035. doi: 10.3390/cells11244035 (PMC9776984; doi:10.3390/cells11244035)
Supplement: Supplementary file 1 [file cells-11-04035-s001.zip › DEGs and enrichment analysis.zip!/DEGs and enrichment analysis/WT_VIMvsVector KEGG/UP/WT_VIMvsVector_up.bar.pdf]

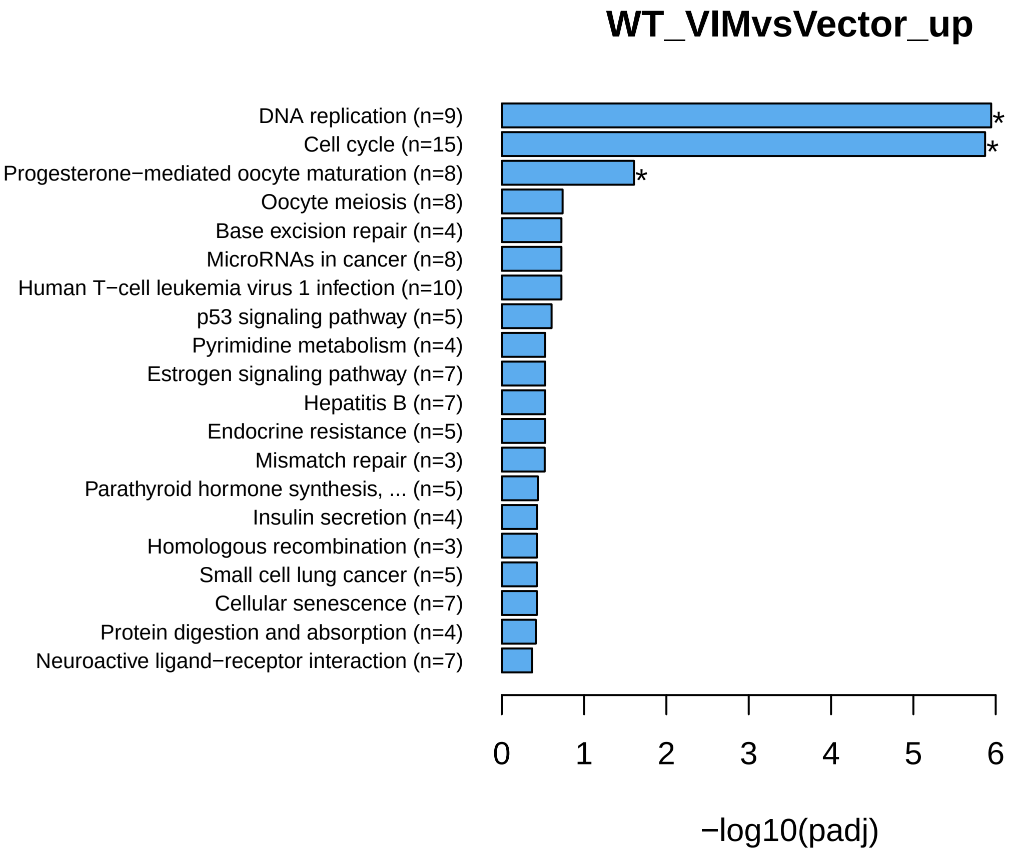

WT_VIMvsVector_up
DNA replication (n=9)
Cell cycle (n=15)
Progesterone−mediated oocyte maturation (n=8)
Oocyte meiosis (n=8)
Base excision repair (n=4)
MicroRNAs in cancer (n=8)
Human T−cell leukemia virus 1 infection (n=10)
p53 signaling pathway (n=5)
Pyrimidine metabolism (n=4)
Estrogen signaling pathway (n=7)
Hepatitis B (n=7)
Endocrine resistance (n=5)
Mismatch repair (n=3)
Parathyroid hormone synthesis, ... (n=5)
Insulin secretion (n=4)
Homologous recombination (n=3)
Small cell lung cancer (n=5)
Cellular senescence (n=7)
Protein digestion and absorption (n=4)
Neuroactive ligand−receptor interaction (n=7)
*
*
*
0
1
2
3
4
5
6
−log10(padj)
